# Decision-Making and Management in a Patient With Coexistent Colloid Cyst and Pituitary Macroadenoma: A Case Report

**DOI:** 10.7759/cureus.22884

**Published:** 2022-03-06

**Authors:** Grant Koskay, Patrick Opperman, Frank M Mezzacappa, Daniel Surdell

**Affiliations:** 1 Neurological Surgery, Creighton University School of Medicine, Omaha, USA; 2 Neurological Surgery, University of Nebraska Medical Center, Omaha, USA

**Keywords:** minimally invasive, tubular retractor, endonasal endoscopic transsphenoidal surgery, surgical decision-making, coexisting brain tumors, pituitary macroadenoma, colloid cyst

## Abstract

The coexistence of separate and distinct primary intracranial tumors is rare. Specifically, there are no previous reports of a colloid cyst coexisting with a pituitary macroadenoma. We present the case of a 40-year-old male with a colloid cyst associated with mild enlargement of the right lateral ventricle and a coexistent pituitary macroadenoma with compression of the optic apparatus. An endoscopic endonasal transsphenoidal surgery (EETS) for resection of the pituitary mass was performed first due to the patient’s complaints of acute visual changes. He then underwent a right frontal craniotomy for resection of the colloid cyst one month later. The patient recovered without residual deficits in vision, and he did not require ventricular shunting after removal of the colloid cyst. We aimed to discuss our decision-making process and the management of these coexistent lesions.

## Introduction

Colloid cysts are benign fluid-filled tumors that typically arise within the third ventricle of the brain and comprise approximately 0.5%-2.0% of all intracranial tumors, with peak incidence occurring between 30-60 years of age [1]. These tumors contain an outer thin fibrous layer of collagen and an inner layer of epithelium that produces a gel-like substance called colloid [1,2]. Colloid cysts may present with hydrocephalus due to obstruction of the normal CSF outflow pathways at the foramen of Monro; however, they are also commonly incidentally found when imaging is performed for another indication such as trauma. Pituitary adenomas typically arise from cells of the adenohypophysis and are classified as microadenoma or macroadenoma based on size less than or greater than 10mm, respectively. Pituitary adenomas are typically benign lesions commonly presenting with new or worsening visual deficit secondary to compression of the optic apparatus [3,4]. The incidence of these tumors can be as high as 94 per 100,000, making up approximately 15% of primary intracranial tumors. Coexistent primary intracranial tumors are rare, and there are no previously reported cases of a coexistent colloid cyst and pituitary macroadenoma, to the best of our knowledge. In this report, we aimed to present and discuss the decision-making process for and surgical management of a patient presenting with a coexistent colloid cyst and pituitary macroadenoma due to the rarity of this clinical scenario and lack of data to help guide neurosurgeons and other practitioners that may encounter similar patients.

## Case presentation

**Figure 1:**
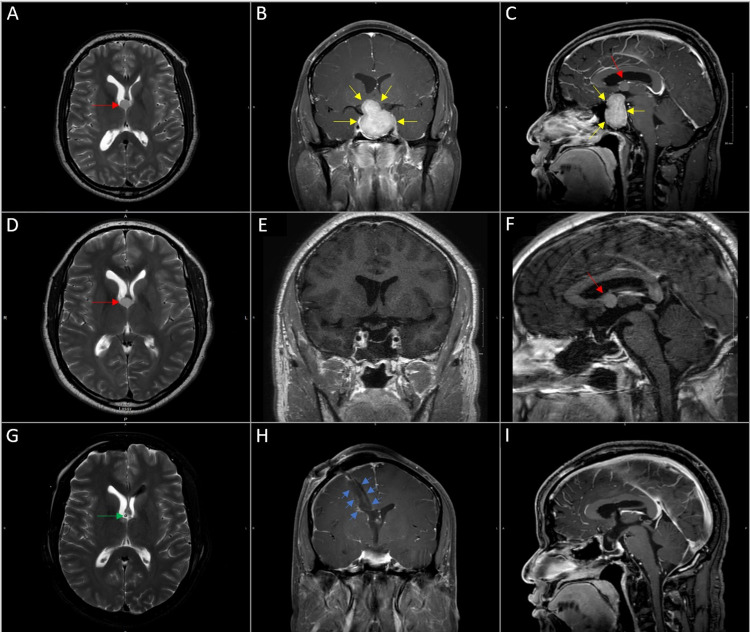
Coexistent colloid cyst and pituitary macroadenoma. MRI of the brain with and without contrast at presentation demonstrates the colloid cyst (red arrows) on an axial T2 sequence with slight enlargement of the frontal horn of the right lateral ventricle but without overt evidence of acute hydrocephalus such as transependymal edema (A), the pituitary macroadenoma (yellow arrows) on a coronal post-contrast T1 sequence (B), and coexistent lesions on a sagittal post-contrast T1 sequence (C). An MRI of the sella with and without contrast after resection of the pituitary macroadenoma demonstrates the stable appearance of the colloid cyst on an axial T2 sequence and sagittal post-contrast T1 sequence (D,F) with evidence of residual macroadenoma around the cavernous sinus bilaterally (E,F). The optic apparatus appeared well-decompressed after EETS of the pituitary macroadenoma (E). An MRI of the brain with and without contrast after subsequent resection of the colloid cyst utilizing the BrainPath® tubular retractor system demonstrates gross total resection with the placement of a temporary ventriculostomy catheter (tip of catheter denoted by the green arrow) in an axial T2 sequence (G) and a coronal post-contrast T1 sequence (H). A sagittal post-contrast T1 image demonstrating both areas of resection simultaneously (I). A small transcortical tract (blue arrows) on a trajectory towards the foramen of Monro demonstrates the minimally invasive access to the right lateral ventricle utilizing a tubular retractor (H).

The patient is a 40-year-old male who presented to the emergency department with three days of progressive worsening in vision. He reported that he was also having a persistent, unrelieved headache for the past month. Bedside examination revealed temporal field deficits. Given the patient’s symptom profile, an MRI of the brain with and without contrast was performed. This demonstrated a well-circumscribed lesion measuring 1 cm in diameter at the foramen of Monro consistent with the appearance of a colloid cyst as well as a 3.9 x 3.5 x 2.2 cm sellar/suprasellar mass with compression of the optic apparatus and likely involvement of the cavernous sinus (Figures 1A-1C). We had a discussion with the patient regarding this unique clinical scenario. Ultimately, we agreed to treat the pituitary lesion first due to the size of the lesion with new-onset visual difficulties and lack of overt hydrocephalus related to the colloid cyst.

The patient underwent endoscopic endonasal transsphenoidal surgery (EETS) for resection of the pituitary macroadenoma. There was no cerebrospinal fluid (CSF) leak noted intraoperatively. A multi-layered closure technique incorporating Surgifoam® (Ethicon Inc., Bridgewater, New Jersey) layered in the sella, covered with the collagen-matrix dural substitute DuraGen® (Integra LifeSciences Co., Princeton, New Jersey) with the patient's dural leaflets reflected superficially over this, followed by a nasal mucosal graft, and finally covered with the fibrin sealant Tisseel (Baxter International Inc., Deerfield, Illinois) was utilized to close the sellar opening to provide multiple layers of protection against any occult cerebrospinal fluid (CSF) leak not visualized during surgery. The patient was transferred to the neuroscience intensive care unit (ICU) for postoperative neurologic and hemodynamic monitoring. An MRI of the sella with and without contrast was performed on a postoperative day one and demonstrated interval subtotal resection of the pituitary macroadenoma with a residual tumor near the cavernous sinus bilaterally (Figures 1D-1F). Laboratory evaluation in the postoperative period identified pertinent hypothalamic-pituitary-adrenal axis (HPA) findings as follows: thyroid-stimulating hormone 2.08 mcIU/mL (nl. range 0.40-4.30 mcIU/mL) with free T4 0.3 ng/dL (nl. range 0.6-1.6 ng/dL) and 8 am cortisol 1.3 mcg/dL (nl. range 6.7-22.6 mcg/dL), which required supplementation of levothyroxine and hydrocortisone. Other pertinent negative laboratory evaluations included: prolactin 15.3 ng/mL (nl. range 2.6-13.1 ng/mL) from mild stalk effect, follicle-stimulating hormone 2.1 mIU/mL (nl. range 1.3-19.3 mIU/mL), luteinizing hormone 1.3 mIU/mL (nl. range 1.3-8.6 mIU/mL), adrenocorticotropic hormone 16 pg/mL (nl. range 0-46 pg/mL), and insulin-like growth factor-1 62 ng/mL (nl. range 48-292 ng/mL). The otolaryngology team performed a bedside stress test before patient mobilization to ensure adequate closure of the sella, again due to the possibility of an occult CSF leak that was not recognized intraoperatively. This test was negative for evidence of a CSF leak, and the patient was mobilized. The patient was then transferred to the floor, had an uneventful course, and was discharged. The final pathology was consistent with a gonadotroph secreting pituitary macroadenoma.

The patient returned to the hospital approximately four weeks after recovery from the first surgery. At this time, the patient underwent a right-sided craniotomy with intraoperative image guidance. A minimally invasive trans-sulcal technique utilizing the BrainPath® (NICO Corp, Indianapolis, Indiana) tubular retractor system allowed access into the frontal horn of the right lateral ventricle with ultimate resection of the colloid cyst, fenestration of the septum pellucidum, and placement of an external ventricular drain (EVD). A postoperative MRI of the brain with and without contrast demonstrated gross total resection of the colloid cyst (Figures 1G-1I). In the first 24 hours postoperatively, he was noted to have difficulties with short-term memory loss. Over the next few days, the patient’s EVD was weaned via the successive increase in drainage pressure required to drain fluid through the system, concluding with a 24-hour EVD clamp trial. He tolerated this wean without the development of new symptoms and; therefore, the EVD was removed. His short-term memory loss improved until he reached his baseline at discharge on postoperative day three. The final pathology was consistent with a colloid cyst. 

The patient has since followed up in the clinic and is doing well from both a neurosurgical and otolaryngological perspective. Outpatient evaluation by ophthalmology demonstrated resolution of the patient’s preoperative visual symptoms. He continues to be followed in the endocrinology clinic.

## Discussion

Colloid cysts are uncommon, slow-growing, benign intracranial lesions that are derived from endoderm [1]. They typically arise within the third ventricle near the foramen of Monro but have also been found in other locations, including the frontal and parietal lobes, the septum pellucidum, fourth ventricle, cerebellum, brainstem, suprasellar region, and others. Colloid cysts are more common in males than in females by a slight ratio of 1.4:1 and usually are found between the third and sixth decades. In one meta-analysis, the mean age at presentation was 40 years, which aligns with our patient [5]. The prevalence of these lesions typically encompasses less than 2% of all intracranial tumors [1]. Colloid cysts have a collagen wall with a layer of epithelium filled with mucinous contents, generally called colloid, that can vary in color [1,2]. Typically, headache is the most common symptom associated with colloid cysts and occurs in about 60% to 90% of patients, and is typically related to CSF outflow obstruction at the foramen of Monro, resulting in hydrocephalus. However, some colloid cysts are identified on imaging performed for a separate indication, such as trauma. Radiologically, colloid cysts typically appear as well-demarcated cystic lesions at the foramen of Monro. Treatment for symptomatic colloid cysts requires surgical resection to prevent neurological deterioration [2]. Difficulty can arise as critical neurovascular structures such as the fornices and internal cerebral veins are typically near to colloid cysts. Open microsurgical and endoscopic techniques have been described [5-9]. Although there have been several reports demonstrating fewer complications associated with endoscopy and better short-term outcomes, a meta-analysis on both of these procedures demonstrated a complete resection rate of 96.8%, favoring open microsurgery vs. 58.2% with endoscopy [6]. The re-operation rate also favored open microsurgery at 0.38% compared with 3% for endoscopy. This suggests that open microsurgery could have better long-term outcomes compared to endoscopy as there is less recurrence. However, controversy regarding the optimal surgical approach remains, and the appropriate technique for an individual lesion should be based on the surgeon’s experience and comfort.

Pituitary adenomas are primary tumors arising from the pituitary gland. They typically arise from cells in the adenohypophysis [10]. Modern classification of these tumors includes evaluation of size, immunohistochemistry, electron microscopy, and functionality based on hormonal activity [11]. Lesions that are smaller than 10 mm are defined as microadenoma, and lesions larger than 10 mm are defined as macroadenomas. The incidence of pituitary adenomas is approximately 3.9 to 7.4 cases per 100,000 per year [4]. The prevalence of these tumors is approximately 1 case per 1,000 in the general population [4,12]. Functional pituitary adenomas most commonly secrete growth hormone, prolactin, or thyroid-stimulating hormone, although they rarely may secrete adrenocorticotropic hormone (ACTH) [13,14]. Pituitary adenomas may also secrete multiple hormones simultaneously, possibly resulting from neoplastic transformation of different cell lines or trans-differentiation of one cell line into a different hormone-producing one. Clinical presentation is variable and may be related to the specific hormone secreted by an individual tumor. For example, these tumors may cause hyperthyroidism, Cushing’s disease, hyperprolactinemia, and acromegaly. Headache and visual changes are common symptoms of pituitary adenomas as these tumors may result in elevations in intracranial pressure as a space-occupying lesion and compression of the optic apparatus, respectively [15]. Surgical intervention is frequently required for symptomatic lesions. The EETS approach is most common due to its less traumatic route and excellent visualization of the sellar region [16-18].

We reviewed the literature in an attempt to find previously reported cases of patients with coexistent colloid cysts and pituitary macroadenomas. We queried PubMed and Google scholar utilizing the terms “colloid cyst”, “pituitary adenoma”, “pituitary macroadenoma”, “dual”, and “coexistent” in various combinations and did not identify any previously reported cases. To the best of our knowledge, our case represents the first report of such a patient. Therefore, the decision-making process regarding treatment staging presented a unique clinical challenge. In this case, the pituitary macroadenoma was resected first due to the patient's acute onset of vision changes and concern for progressive or permanent visual loss. Although the patient did have headaches, this symptom was stable for one month, and there was a lack of transependymal flow of CSF on imaging to suggest overt hydrocephalus related to the colloid cyst. Thus, we felt it was safe to proceed with resection of the pituitary macroadenoma first with close follow-up and resection of the colloid cyst after recovery from the initial surgery. Furthermore, the coexistent pituitary macroadenoma did not affect the choice of approach to the colloid cyst in our case since we were able to resect the pituitary lesion via the EETS route. We felt that the use of the tubular retractor system was most appropriate for the colloid cyst due to prior experience with the technique, ability to perform a smaller craniotomy in a patient with recent major surgery, and an enlarged lateral ventricle allowing for adequate maneuverability within the ventricular system through the tubular retractor. Importantly, there was no CSF leak related to the EETS. A CSF leak would have resulted in more frequent postoperative cranial imaging and a lower threshold to drain CSF via lumbar drainage. The consequences of draining CSF in the setting of a colloid cyst are unknown, but there is concern that a change in the CSF fluid dynamics in such a patient could result in the acute onset of obstructive hydrocephalus. Therefore, earlier resection of the colloid cyst in our case may have been necessary if there were radiographic or clinical changes related to the management of complications after an EETS.

The specific procedure staging presented here may not apply to other patients with coexisting colloid cysts and pituitary macroadenomas. For example, a patient may present primarily with headache and imaging evidence of hydrocephalus in the setting of CSF outflow obstruction by a colloid cyst that is incidentally noted to have an asymptomatic pituitary macroadenoma. The colloid cyst is the more symptomatic lesion in that scenario and should be treated first. Multiple factors should be considered in the decision-making process, such as acuity of symptoms, symptom relation to individual lesions, size of the lesions, the likely diagnosis of each lesion based on clinical and radiological information, and the patient’s comorbid status and ability to tolerate multiple cranial surgeries in a short period. Therefore, we cannot make formal recommendations regarding the most appropriate timing for the surgical management of coexistent colloid cyst and pituitary macroadenoma. However, we hope this presentation and discussion provide a framework for neurosurgeons and other practitioners regarding the thought process involved in decision-making for patients with similar findings.

## Conclusions

Coexistent primary intracranial tumors are rare. There are no previously reported cases of the coexistent colloid cyst and pituitary macroadenoma. The decision-making process for such a patient is complex due to the possibility of neurological compromise from both lesions and the lack of previous reports and data to help guide management. Ultimately, the lesion that is felt to provide the highest acute risk of neurological compromise should be treated first.
